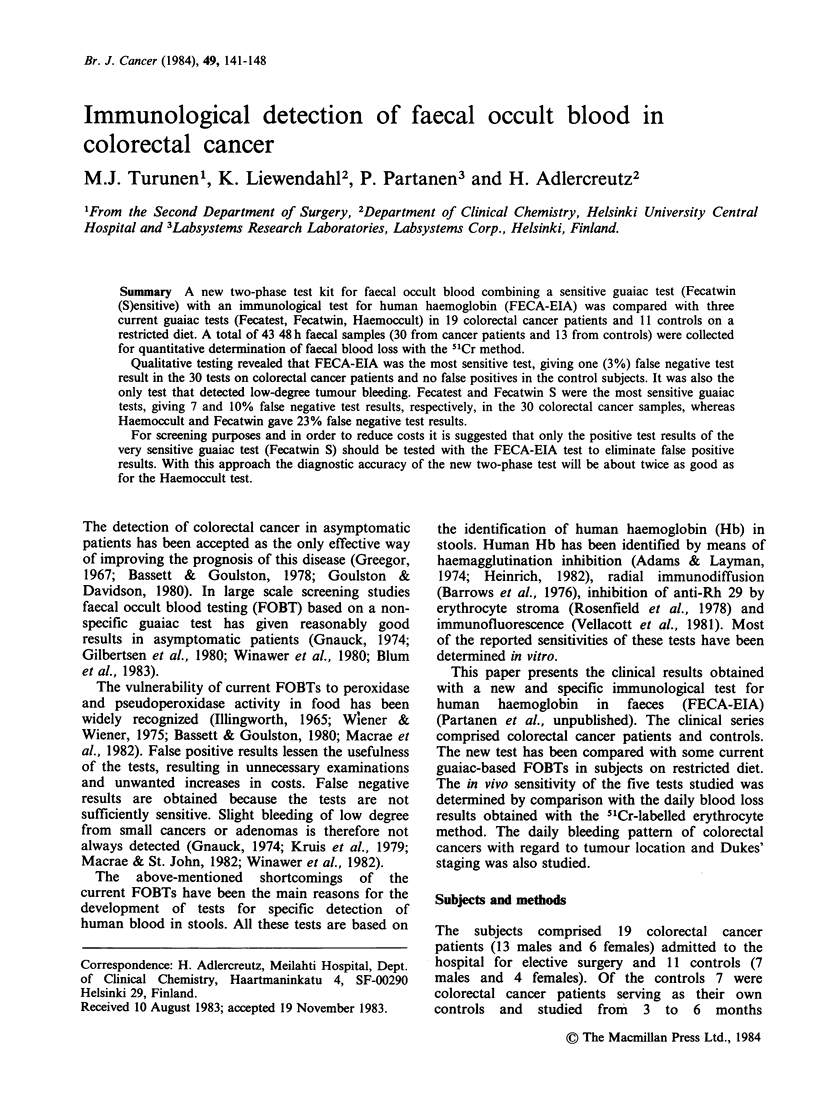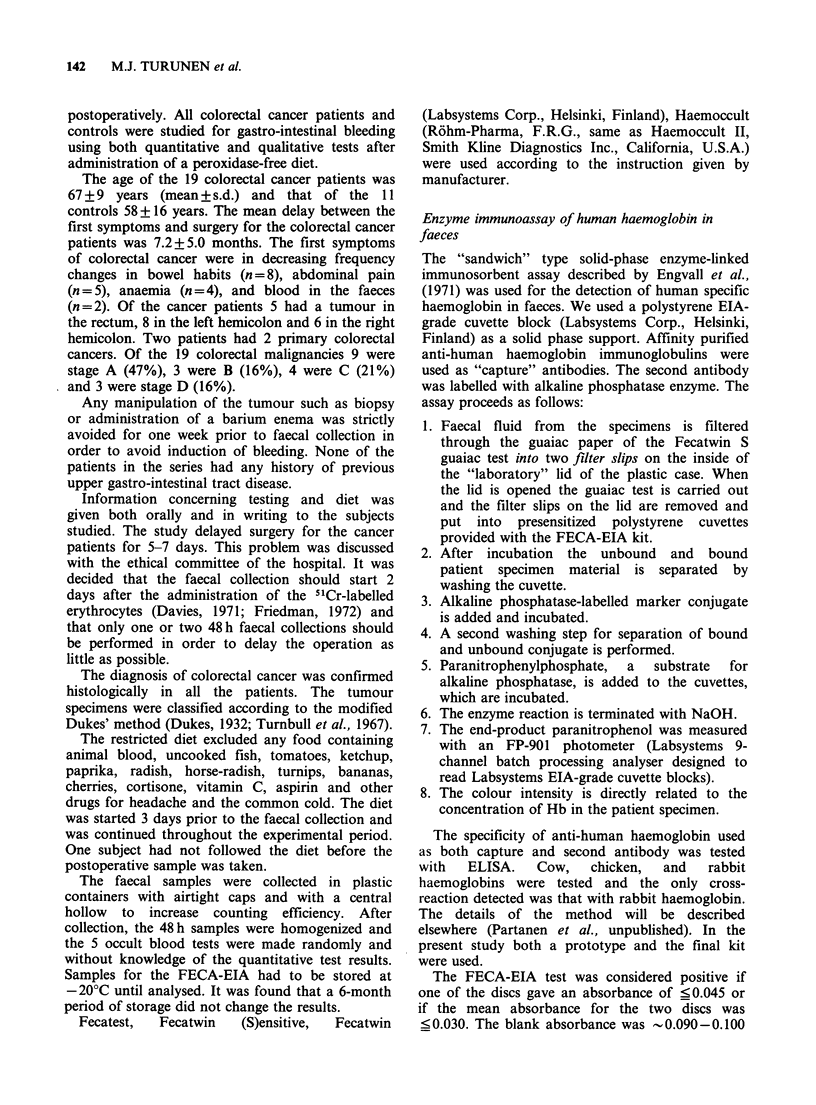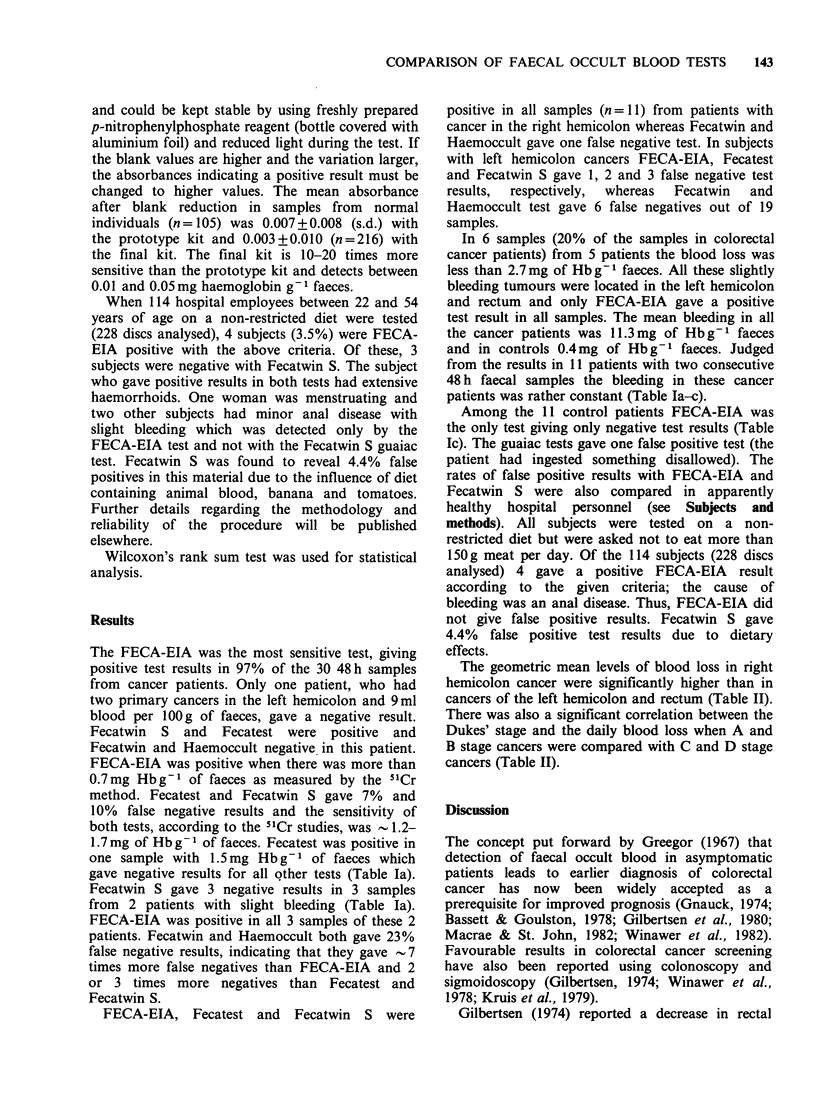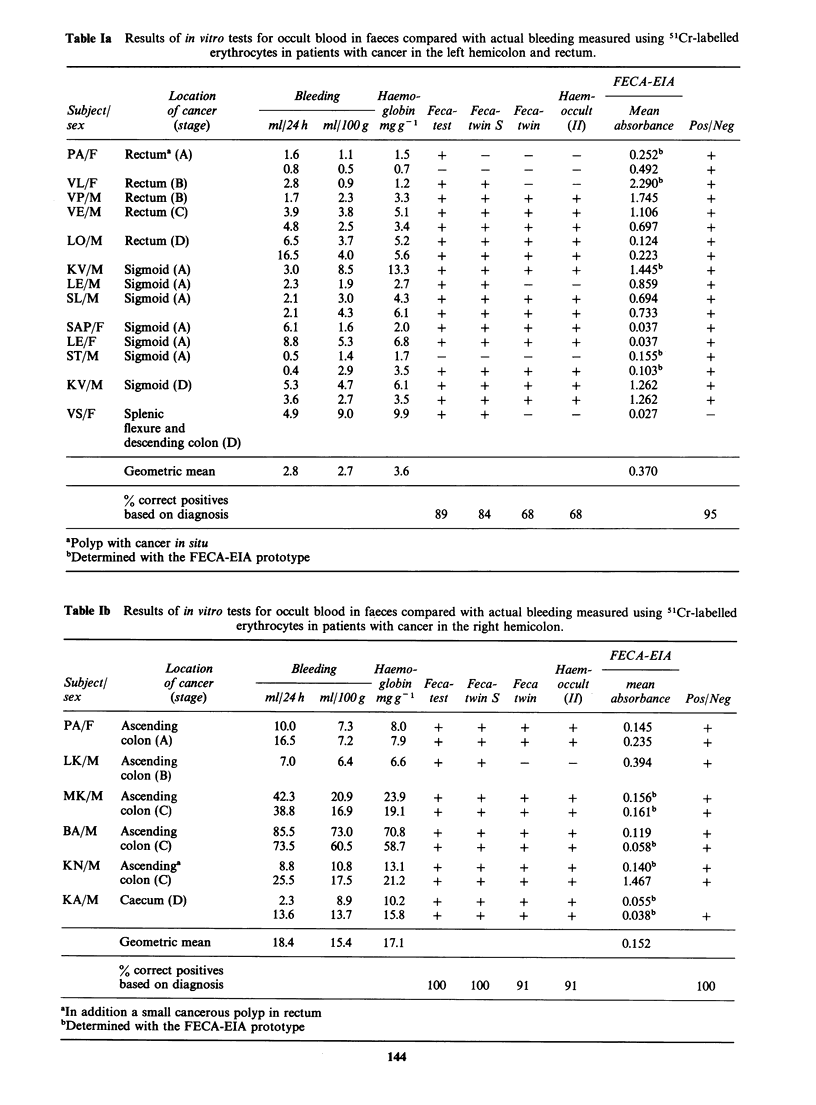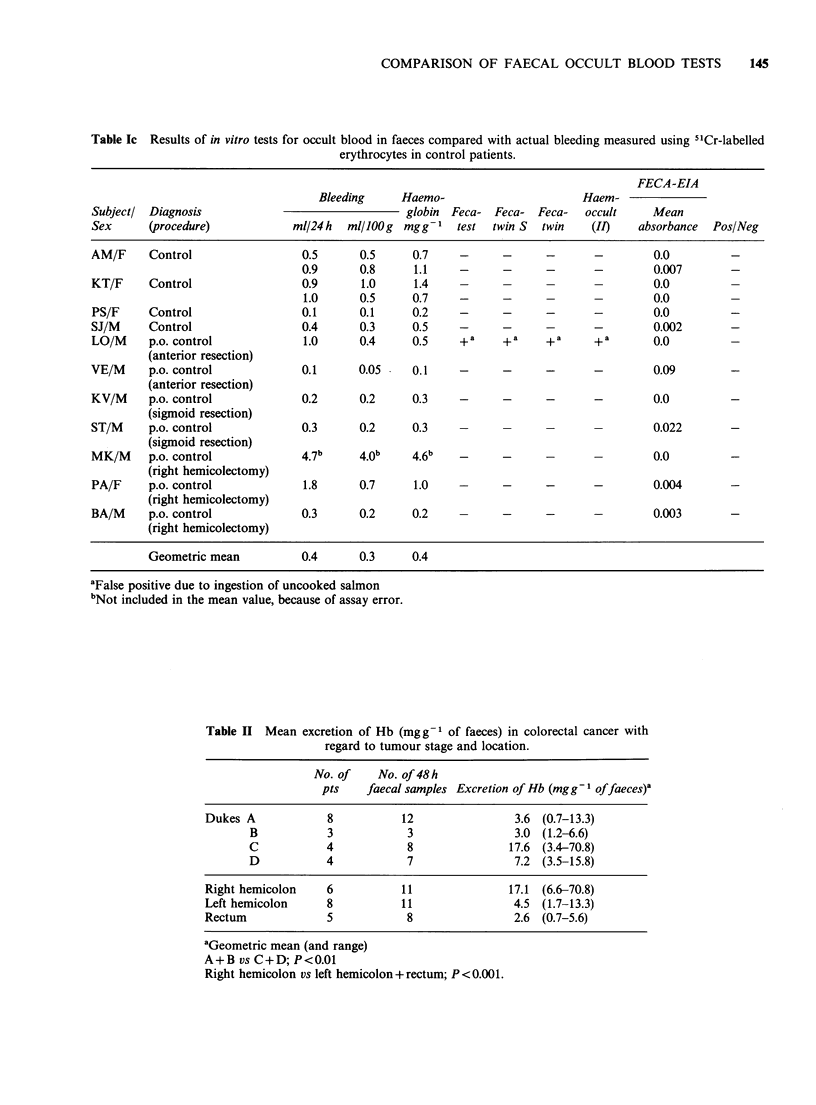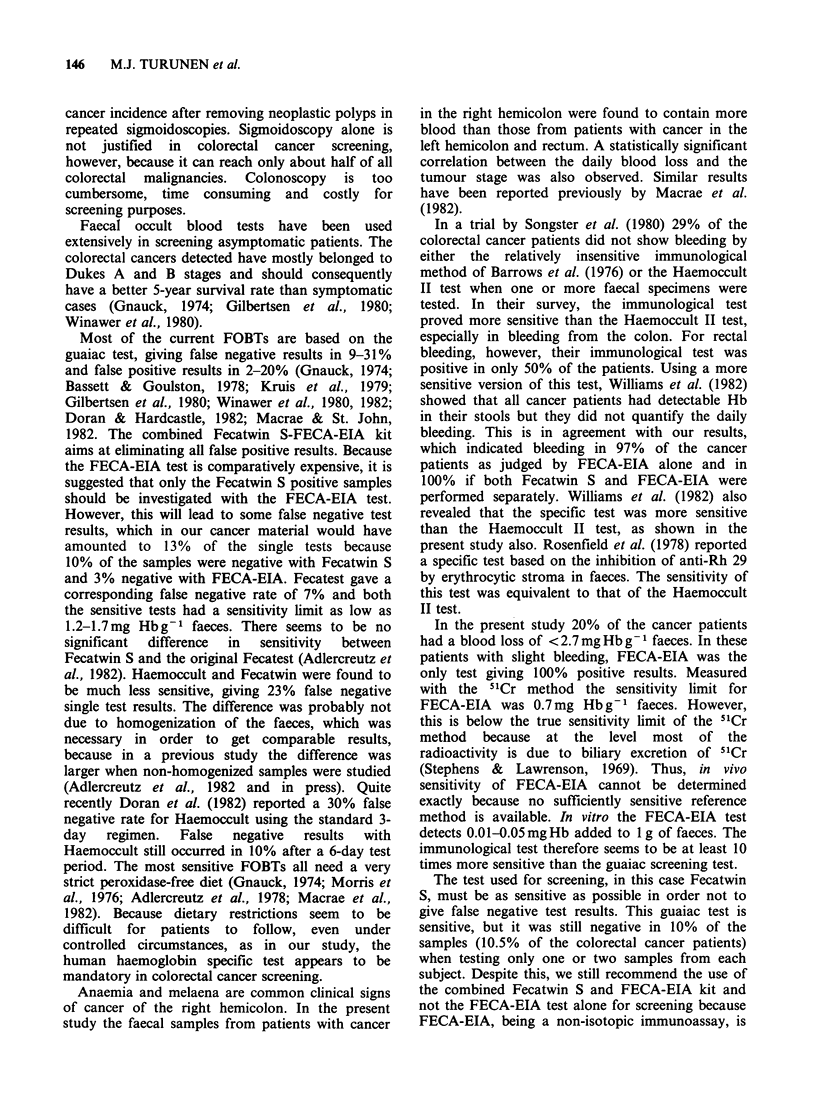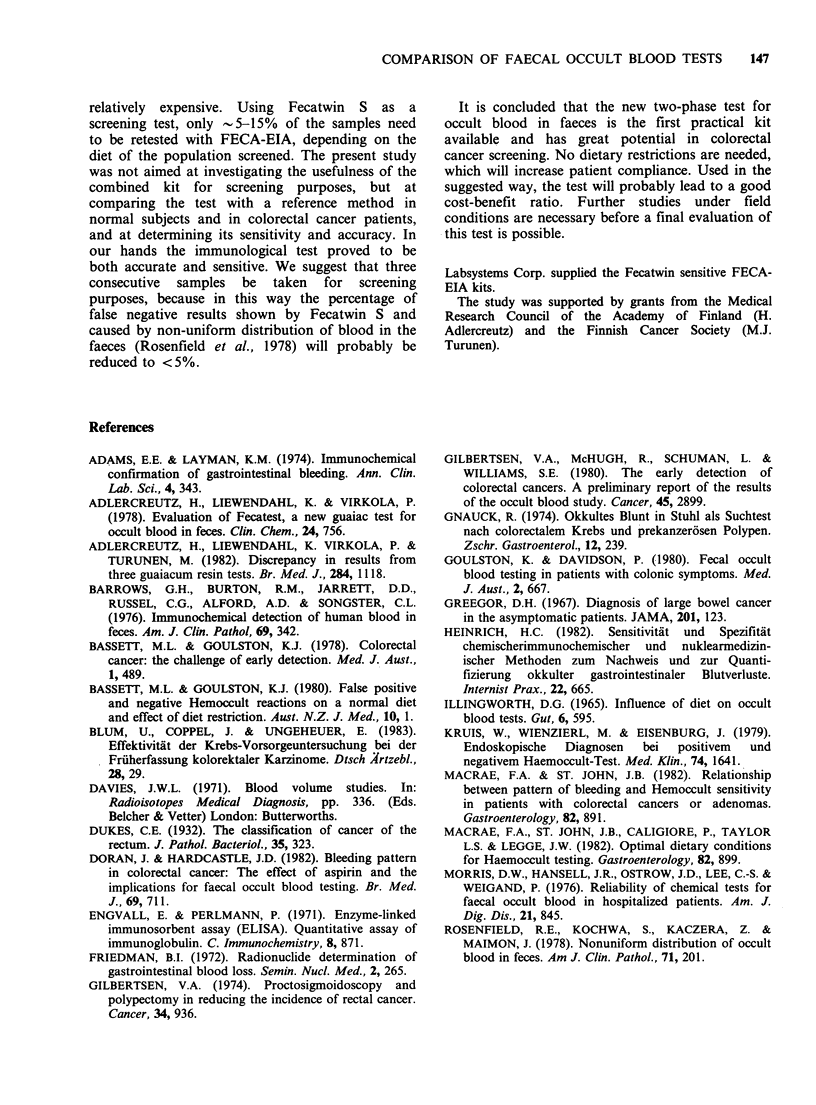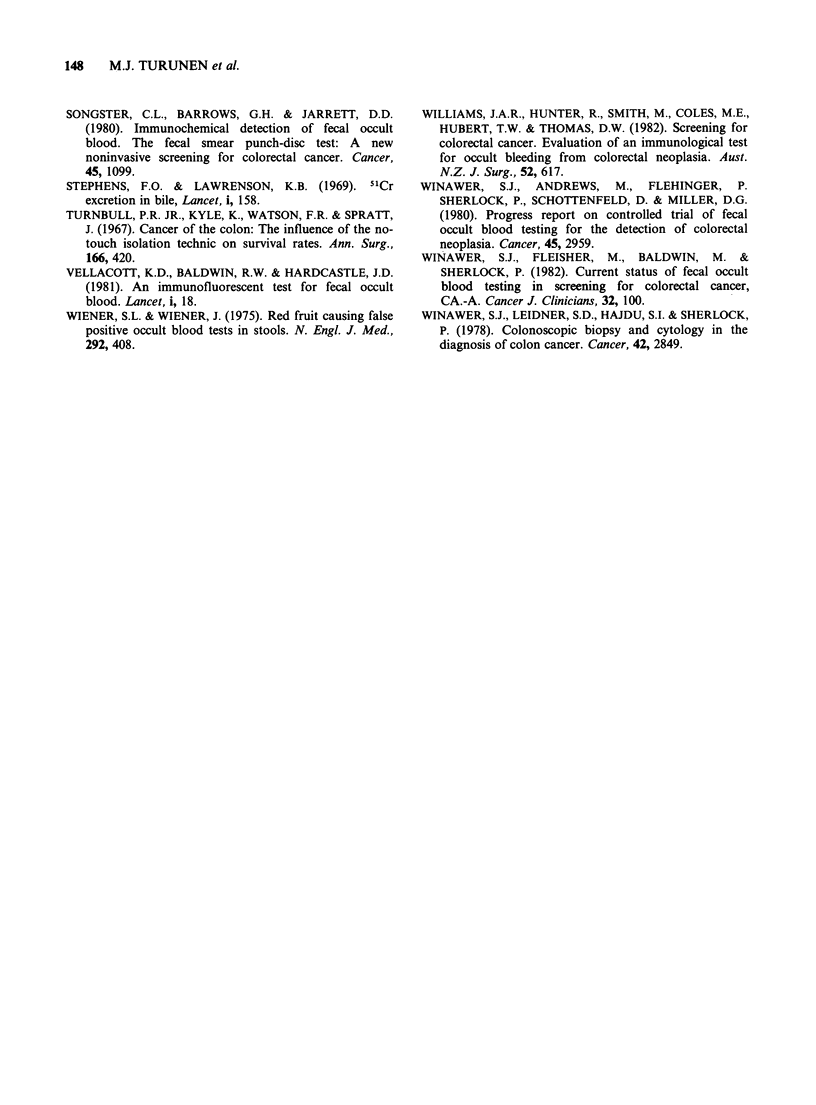# Immunological detection of faecal occult blood in colorectal cancer.

**DOI:** 10.1038/bjc.1984.26

**Published:** 1984-02

**Authors:** M. J. Turunen, K. Liewendahl, P. Partanen, H. Adlercreutz

## Abstract

A new two-phase test kit for faecal occult blood combining a sensitive guaiac test (Fecatwin (S)ensitive) with an immunological test for human haemoglobin (FECA-EIA) was compared with three current guaiac tests (Fecatest, Fecatwin, Haemoccult) in 19 colorectal cancer patients and 11 controls on a restricted diet. A total of 43 48 h faecal samples (30 from cancer patients and 13 from controls) were collected for quantitative determination of faecal blood loss with the 51Cr method. Qualitative testing revealed that FECA-EIA was the most sensitive test, giving one (3%) false negative test result in the 30 tests on colorectal cancer patients and no false positives in the control subjects. It was also the only test that detected low-degree tumour bleeding. Fecatest and Fecatwin S were the most sensitive guaiac tests, giving 7 and 10% false negative test results, respectively, in the 30 colorectal cancer samples, whereas Haemoccult and Fecatwin gave 23% false negative test results. For screening purposes and in order to reduce costs it is suggested that only the positive test results of the very sensitive guaiac test (Fecatwin S) should be tested with the FECA-EIA test to eliminate false positive results. With this approach the diagnostic accuracy of the new two-phase test will be about twice as good as for the Haemoccult test.


*Br. J. Cancer* (1984), **49**, 141-148

### Immunological detection of faecal occult blood in colorectal cancer

M.J. Turunen[1], K. Liewendahl[2], P. Partanen[3] and H. Adlercreutz[2]

[1]*From the Second Department of Surgery,* [2]*Department of Clinical Chemistry, Helsinki University Central Hospital and* [3]*Labsystems Research Laboratories, Labsystems Corp., Helsinki, Finland.*

**Summary** A new two-phase test kit for faecal occult blood combining a sensitive guaiac test (Fecatwin (S)ensitive) with an immunological test for human haemoglobin (FECA-EIA) was compared with three current guaiac tests (Fecatest, Fecatwin, Haemoccult) in 19 colorectal cancer patients and 11 controls on a restricted diet. A total of 43 48 h faecal samples (30 from cancer patients and 13 from controls) were collected for quantitative determination of faecal blood loss with the $^{51}Cr$ method.

Qualitative testing revealed that FECA-EIA was the most sensitive test, giving one (3%) false negative test result in the 30 tests on colorectal cancer patients and no false positives in the control subjects. It was also the only test that detected low-degree tumour bleeding. Fecatest and Fecatwin S were the most sensitive guaiac tests, giving 7 and 10% false negative test results, respectively, in the 30 colorectal cancer samples, whereas Haemoccult and Fecatwin gave 23% false negative test results.

For screening purposes and in order to reduce costs it is suggested that only the positive test results of the very sensitive guaiac test (Fecatwin S) should be tested with the FECA-EIA test to eliminate false positive results. With this approach the diagnostic accuracy of the new two-phase test will be about twice as good as for the Haemoccult test.

The detection of colorectal cancer in asymptomatic patients has been accepted as the only effective way of improving the prognosis of this disease (Greegor, 1967; Bassett & Goulston, 1978; Goulston & Davidson, 1980). In large scale screening studies faecal occult blood testing (FOBT) based on a non-specific guaiac test has given reasonably good results in asymptomatic patients (Gnauck, 1974; Gilbertsen *et al.*, 1980; Winawer *et al.*, 1980; Blum *et al.*, 1983).

The vulnerability of current FOBTs to peroxidase and pseudoperoxidase activity in food has been widely recognized (Illingworth, 1965; Wiener & Wiener, 1975; Bassett & Goulston, 1980; Macrae *et al.*, 1982). False positive results lessen the usefulness of the tests, resulting in unnecessary examinations and unwanted increases in costs. False negative results are obtained because the tests are not sufficiently sensitive. Slight bleeding of low degree from small cancers or adenomas is therefore not always detected (Gnauck, 1974; Kruis *et al.*, 1979; Macrae & St. John, 1982; Winawer *et al.*, 1982).

The above-mentioned shortcomings of the current FOBTs have been the main reasons for the development of tests for specific detection of human blood in stools. All these tests are based on the identification of human haemoglobin (Hb) in stools. Human Hb has been identified by means of haemagglutination inhibition (Adams & Layman, 1974; Heinrich, 1982), radial immunodiffusion (Barrows *et al.*, 1976), inhibition of anti-Rh 29 by erythrocyte stroma (Rosenfield *et al.*, 1978) and immunofluorescence (Vellacott *et al.*, 1981). Most of the reported sensitivities of these tests have been determined *in vitro*.

This paper presents the clinical results obtained with a new and specific immunological test for human haemoglobin in faeces (FECA-EIA) (Partanen *et al.*, unpublished). The clinical series comprised colorectal cancer patients and controls. The new test has been compared with some current guaiac-based FOBTs in subjects on restricted diet. The *in vivo* sensitivity of the five tests studied was determined by comparison with the daily blood loss results obtained with the $^{51}Cr$-labelled erythrocyte method. The daily bleeding pattern of colorectal cancers with regard to tumour location and Dukes' staging was also studied.

### Subjects and methods

The subjects comprised 19 colorectal cancer patients (13 males and 6 females) admitted to the hospital for elective surgery and 11 controls (7 males and 4 females). Of the controls 7 were colorectal cancer patients serving as their own controls and studied from 3 to 6 months

Correspondence: H. Adlercreutz, Meilahti Hospital, Dept. of Clinical Chemistry, Haartmaninkatu 4, SF-00290 Helsinki 29, Finland.
Received 10 August 1983; accepted 19 November 1983.

© The Macmillan Press Ltd., 1984

postoperatively. All colorectal cancer patients and controls were studied for gastro-intestinal bleeding using both quantitative and qualitative tests after administration of a peroxidase-free diet.

The age of the 19 colorectal cancer patients was $67 \pm 9$ years (mean $\pm$ s.d.) and that of the 11 controls $58 \pm 16$ years. The mean delay between the first symptoms and surgery for the colorectal cancer patients was $7.2 \pm 5.0$ months. The first symptoms of colorectal cancer were in decreasing frequency changes in bowel habits ($n=8$), abdominal pain ($n=5$), anaemia ($n=4$), and blood in the faeces ($n=2$). Of the cancer patients 5 had a tumour in the rectum, 8 in the left hemicolon and 6 in the right hemicolon. Two patients had 2 primary colorectal cancers. Of the 19 colorectal malignancies 9 were stage A (47%), 3 were B (16%), 4 were C (21%) and 3 were stage D (16%).

Any manipulation of the tumour such as biopsy or administration of a barium enema was strictly avoided for one week prior to faecal collection in order to avoid induction of bleeding. None of the patients in the series had any history of previous upper gastro-intestinal tract disease.

Information concerning testing and diet was given both orally and in writing to the subjects studied. The study delayed surgery for the cancer patients for 5–7 days. This problem was discussed with the ethical committee of the hospital. It was decided that the faecal collection should start 2 days after the administration of the $^{51}$Cr-labelled erythrocytes (Davies, 1971; Friedman, 1972) and that only one or two 48 h faecal collections should be performed in order to delay the operation as little as possible.

The diagnosis of colorectal cancer was confirmed histologically in all the patients. The tumour specimens were classified according to the modified Dukes' method (Dukes, 1932; Turnbull *et al.*, 1967).

The restricted diet excluded any food containing animal blood, uncooked fish, tomatoes, ketchup, paprika, radish, horse-radish, turnips, bananas, cherries, cortisone, vitamin C, aspirin and other drugs for headache and the common cold. The diet was started 3 days prior to the faecal collection and was continued throughout the experimental period. One subject had not followed the diet before the postoperative sample was taken.

The faecal samples were collected in plastic containers with airtight caps and with a central hollow to increase counting efficiency. After collection, the 48 h samples were homogenized and the 5 occult blood tests were made randomly and without knowledge of the quantitative test results. Samples for the FECA-EIA had to be stored at $-20$°C until analysed. It was found that a 6-month period of storage did not change the results.

Fecatest, Fecatwin (S)ensitive, Fecatwin (Labsystems Corp., Helsinki, Finland), Haemoccult (Röhm-Pharma, F.R.G., same as Haemoccult II, Smith Kline Diagnostics Inc., California, U.S.A.) were used according to the instruction given by manufacturer.

### *Enzyme immunoassay of human haemoglobin in faeces*

The "sandwich" type solid-phase enzyme-linked immunosorbent assay described by Engvall *et al.*, (1971) was used for the detection of human specific haemoglobin in faeces. We used a polystyrene EIA-grade cuvette block (Labsystems Corp., Helsinki, Finland) as a solid phase support. Affinity purified anti-human haemoglobin immunoglobulins were used as "capture" antibodies. The second antibody was labelled with alkaline phosphatase enzyme. The assay proceeds as follows:

1. Faecal fluid from the specimens is filtered through the guaiac paper of the Fecatwin S guaiac test *into* two *filter slips* on the inside of the "laboratory" lid of the plastic case. When the lid is opened the guaiac test is carried out and the filter slips on the lid are removed and put into presensitized polystyrene cuvettes provided with the FECA-EIA kit.
2. After incubation the unbound and bound patient specimen material is separated by washing the cuvette.
3. Alkaline phosphatase-labelled marker conjugate is added and incubated.
4. A second washing step for separation of bound and unbound conjugate is performed.
5. Paranitrophenylphosphate, a substrate for alkaline phosphatase, is added to the cuvettes, which are incubated.
6. The enzyme reaction is terminated with NaOH.
7. The end-product paranitrophenol was measured with an FP-901 photometer (Labsystems 9-channel batch processing analyser designed to read Labsystems EIA-grade cuvette blocks).
8. The colour intensity is directly related to the concentration of Hb in the patient specimen.

The specificity of anti-human haemoglobin used as both capture and second antibody was tested with ELISA. Cow, chicken, and rabbit haemoglobins were tested and the only cross-reaction detected was that with rabbit haemoglobin. The details of the method will be described elsewhere (Partanen *et al.*, unpublished). In the present study both a prototype and the final kit were used.

The FECA-EIA test was considered positive if one of the discs gave an absorbance of $\leqq 0.045$ or if the mean absorbance for the two discs was $\leqq 0.030$. The blank absorbance was $\sim 0.090 - 0.100$

and could be kept stable by using freshly prepared *p*-nitrophenylphosphate reagent (bottle covered with aluminium foil) and reduced light during the test. If the blank values are higher and the variation larger, the absorbances indicating a positive result must be changed to higher values. The mean absorbance after blank reduction in samples from normal individuals ($n = 105$) was $0.007 \pm 0.008$ (s.d.) with the prototype kit and $0.003 \pm 0.010$ ($n = 216$) with the final kit. The final kit is 10–20 times more sensitive than the prototype kit and detects between 0.01 and 0.05 mg haemoglobin $g^{-1}$ faeces.

When 114 hospital employees between 22 and 54 years of age on a non-restricted diet were tested (228 discs analysed), 4 subjects (3.5%) were FECA-EIA positive with the above criteria. Of these, 3 subjects were negative with Fecatwin S. The subject who gave positive results in both tests had extensive haemorrhoids. One woman was menstruating and two other subjects had minor anal disease with slight bleeding which was detected only by the FECA-EIA test and not with the Fecatwin S guaiac test. Fecatwin S was found to reveal 4.4% false positives in this material due to the influence of diet containing animal blood, banana and tomatoes. Further details regarding the methodology and reliability of the procedure will be published elsewhere.

Wilcoxon's rank sum test was used for statistical analysis.

### Results

The FECA-EIA was the most sensitive test, giving positive test results in 97% of the 30 48 h samples from cancer patients. Only one patient, who had two primary cancers in the left hemicolon and 9 ml blood per 100 g of faeces, gave a negative result. Fecatwin S and Fecatest were positive and Fecatwin and Haemoccult negative in this patient. FECA-EIA was positive when there was more than 0.7 mg Hb $g^{-1}$ of faeces as measured by the $^{51}Cr$ method. Fecatest and Fecatwin S gave 7% and 10% false negative results and the sensitivity of both tests, according to the $^{51}Cr$ studies, was $\sim$1.2–1.7 mg of Hb $g^{-1}$ of faeces. Fecatest was positive in one sample with 1.5 mg Hb $g^{-1}$ of faeces which gave negative results for all other tests (Table Ia). Fecatwin S gave 3 negative results in 3 samples from 2 patients with slight bleeding (Table Ia). FECA-EIA was positive in all 3 samples of these 2 patients. Fecatwin and Haemoccult both gave 23% false negative results, indicating that they gave $\sim$7 times more false negatives than FECA-EIA and 2 or 3 times more negatives than Fecatest and Fecatwin S.

FECA-EIA, Fecatest and Fecatwin S were positive in all samples ($n = 11$) from patients with cancer in the right hemicolon whereas Fecatwin and Haemoccult gave one false negative test. In subjects with left hemicolon cancers FECA-EIA, Fecatest and Fecatwin S gave 1, 2 and 3 false negative test results, respectively, whereas Fecatwin and Haemoccult test gave 6 false negatives out of 19 samples.

In 6 samples (20% of the samples in colorectal cancer patients) from 5 patients the blood loss was less than 2.7 mg of Hb $g^{-1}$ faeces. All these slightly bleeding tumours were located in the left hemicolon and rectum and only FECA-EIA gave a positive test result in all samples. The mean bleeding in all the cancer patients was 11.3 mg of Hb $g^{-1}$ faeces and in controls 0.4 mg of Hb $g^{-1}$ faeces. Judged from the results in 11 patients with two consecutive 48 h faecal samples the bleeding in these cancer patients was rather constant (Table Ia–c).

Among the 11 control patients FECA-EIA was the only test giving only negative test results (Table Ic). The guaiac tests gave one false positive test (the patient had ingested something disallowed). The rates of false positive results with FECA-EIA and Fecatwin S were also compared in apparently healthy hospital personnel (see **Subjects and methods**). All subjects were tested on a non-restricted diet but were asked not to eat more than 150 g meat per day. Of the 114 subjects (228 discs analysed) 4 gave a positive FECA-EIA result according to the given criteria; the cause of bleeding was an anal disease. Thus, FECA-EIA did not give false positive results. Fecatwin S gave 4.4% false positive test results due to dietary effects.

The geometric mean levels of blood loss in right hemicolon cancer were significantly higher than in cancers of the left hemicolon and rectum (Table II). There was also a significant correlation between the Dukes' stage and the daily blood loss when A and B stage cancers were compared with C and D stage cancers (Table II).

### Discussion

The concept put forward by Greegor (1967) that detection of faecal occult blood in asymptomatic patients leads to earlier diagnosis of colorectal cancer has now been widely accepted as a prerequisite for improved prognosis (Gnauck, 1974; Bassett & Goulston, 1978; Gilbertsen *et al.*, 1980; Macrae & St. John, 1982; Winawer *et al.*, 1982). Favourable results in colorectal cancer screening have also been reported using colonoscopy and sigmoidoscopy (Gilbertsen, 1974; Winawer *et al.*, 1978; Kruis *et al.*, 1979).

Gilbertsen (1974) reported a decrease in rectal

**Table Ia** Results of *in vitro* tests for occult blood in faeces compared with actual bleeding measured using $^{51}$Cr-labelled erythrocytes in patients with cancer in the left hemicolon and rectum.

| *Subject/ sex* | *Location of cancer (stage)* | *Bleeding* | | *Haemo- globin* | *Feca-* | *Feca-* | *Feca-* | *Haem- occult* | *FECA-EIA* | |
|---|---|---|---|---|---|---|---|---|---|---|
| | | *ml/24 h* | *ml/100 g* | *mg g$^{-1}$* | *test* | *twin S* | *twin* | *(II)* | *Mean absorbance* | *Pos/Neg* |
| PA/F | Rectum[a] (A) | 1.6 | 1.1 | 1.5 | + | – | – | – | 0.252[b] | + |
| | | 0.8 | 0.5 | 0.7 | – | – | – | – | 0.492 | + |
| VL/F | Rectum (B) | 2.8 | 0.9 | 1.2 | + | + | – | – | 2.290[b] | + |
| VP/M | Rectum (B) | 1.7 | 2.3 | 3.3 | + | + | + | + | 1.745 | + |
| VE/M | Rectum (C) | 3.9 | 3.8 | 5.1 | + | + | + | + | 1.106 | + |
| | | 4.8 | 2.5 | 3.4 | + | + | + | + | 0.697 | + |
| LO/M | Rectum (D) | 6.5 | 3.7 | 5.2 | + | + | + | + | 0.124 | + |
| | | 16.5 | 4.0 | 5.6 | + | + | + | + | 0.223 | + |
| KV/M | Sigmoid (A) | 3.0 | 8.5 | 13.3 | + | + | + | + | 1.445[b] | + |
| LE/M | Sigmoid (A) | 2.3 | 1.9 | 2.7 | + | + | – | – | 0.859 | + |
| SL/M | Sigmoid (A) | 2.1 | 3.0 | 4.3 | + | + | + | + | 0.694 | + |
| | | 2.1 | 4.3 | 6.1 | + | + | + | + | 0.733 | + |
| SAP/F | Sigmoid (A) | 6.1 | 1.6 | 2.0 | + | + | + | + | 0.037 | + |
| LE/F | Sigmoid (A) | 8.8 | 5.3 | 6.8 | + | + | + | + | 0.037 | + |
| ST/M | Sigmoid (A) | 0.5 | 1.4 | 1.7 | – | – | – | – | 0.155[b] | + |
| | | 0.4 | 2.9 | 3.5 | + | + | + | + | 0.103[b] | + |
| KV/M | Sigmoid (D) | 5.3 | 4.7 | 6.1 | + | + | + | + | 1.262 | + |
| | | 3.6 | 2.7 | 3.5 | + | + | + | + | 1.262 | + |
| VS/F | Splenic flexure and descending colon (D) | 4.9 | 9.0 | 9.9 | + | + | – | – | 0.027 | – |
| | Geometric mean | 2.8 | 2.7 | 3.6 | | | | | 0.370 | |
| | % correct positives based on diagnosis | | | | 89 | 84 | 68 | 68 | | 95 |

[a]Polyp with cancer *in situ*
[b]Determined with the FECA-EIA prototype

**Table Ib** Results of *in vitro* tests for occult blood in faeces compared with actual bleeding measured using $^{51}$Cr-labelled erythrocytes in patients with cancer in the right hemicolon.

| *Subject/ sex* | *Location of cancer (stage)* | *Bleeding* | | *Haemo- globin* | *Feca-* | *Feca-* | *Feca* | *Haem- occult* | *FECA-EIA* | |
|---|---|---|---|---|---|---|---|---|---|---|
| | | *ml/24 h* | *ml/100 g* | *mg g$^{-1}$* | *test* | *twin S* | *twin* | *(II)* | *mean absorbance* | *Pos/Neg* |
| PA/F | Ascending colon (A) | 10.0 | 7.3 | 8.0 | + | + | + | + | 0.145 | + |
| | | 16.5 | 7.2 | 7.9 | + | + | + | + | 0.235 | + |
| LK/M | Ascending colon (B) | 7.0 | 6.4 | 6.6 | + | + | – | – | 0.394 | + |
| MK/M | Ascending colon (C) | 42.3 | 20.9 | 23.9 | + | + | + | + | 0.156[b] | + |
| | | 38.8 | 16.9 | 19.1 | + | + | + | + | 0.161[b] | + |
| BA/M | Ascending colon (C) | 85.5 | 73.0 | 70.8 | + | + | + | + | 0.119 | + |
| | | 73.5 | 60.5 | 58.7 | + | + | + | + | 0.058[b] | + |
| KN/M | Ascending[a] colon (C) | 8.8 | 10.8 | 13.1 | + | + | + | + | 0.140[b] | + |
| | | 25.5 | 17.5 | 21.2 | + | + | + | + | 1.467 | + |
| KA/M | Caecum (D) | 2.3 | 8.9 | 10.2 | + | + | + | + | 0.055[b] | |
| | | 13.6 | 13.7 | 15.8 | + | + | + | + | 0.038[b] | + |
| | Geometric mean | 18.4 | 15.4 | 17.1 | | | | | 0.152 | |
| | % correct positives based on diagnosis | | | | 100 | 100 | 91 | 91 | | 100 |

[a]In addition a small cancerous polyp in rectum
[b]Determined with the FECA-EIA prototype

**Table Ic** Results of *in vitro* tests for occult blood in faeces compared with actual bleeding measured using $^{51}Cr$-labelled erythrocytes in control patients.

| Subject/ Sex | Diagnosis (procedure) | Bleeding ml/24 h | Bleeding ml/100 g | Haemo-globin mg g$^{-1}$ | Feca-test | Feca-twin S | Feca-twin | Haem-occult (II) | FECA-EIA Mean absorbance | FECA-EIA Pos/Neg |
|---|---|---|---|---|---|---|---|---|---|---|
| AM/F | Control | 0.5 | 0.5 | 0.7 | – | – | – | – | 0.0 | – |
| | | 0.9 | 0.8 | 1.1 | – | – | – | – | 0.007 | – |
| KT/F | Control | 0.9 | 1.0 | 1.4 | – | – | – | – | 0.0 | – |
| | | 1.0 | 0.5 | 0.7 | – | – | – | – | 0.0 | – |
| PS/F | Control | 0.1 | 0.1 | 0.2 | – | – | – | – | 0.0 | – |
| SJ/M | Control | 0.4 | 0.3 | 0.5 | – | – | – | – | 0.002 | – |
| LO/M | p.o. control (anterior resection) | 1.0 | 0.4 | 0.5 | +[a] | +[a] | +[a] | +[a] | 0.0 | – |
| VE/M | p.o. control (anterior resection) | 0.1 | 0.05 | 0.1 | – | – | – | – | 0.09 | – |
| KV/M | p.o. control (sigmoid resection) | 0.2 | 0.2 | 0.3 | – | – | – | – | 0.0 | – |
| ST/M | p.o. control (sigmoid resection) | 0.3 | 0.2 | 0.3 | – | – | – | – | 0.022 | – |
| MK/M | p.o. control (right hemicolectomy) | 4.7[b] | 4.0[b] | 4.6[b] | – | – | – | – | 0.0 | – |
| PA/F | p.o. control (right hemicolectomy) | 1.8 | 0.7 | 1.0 | – | – | – | – | 0.004 | – |
| BA/M | p.o. control (right hemicolectomy) | 0.3 | 0.2 | 0.2 | – | – | – | – | 0.003 | – |
| | Geometric mean | 0.4 | 0.3 | 0.4 | | | | | | |

[a]False positive due to ingestion of uncooked salmon
[b]Not included in the mean value, because of assay error.

**Table II** Mean excretion of Hb (mg g$^{-1}$ of faeces) in colorectal cancer with regard to tumour stage and location.

| | No. of pts | No. of 48 h faecal samples | Excretion of Hb (mg g$^{-1}$ of faeces)[a] |
|---|---|---|---|
| Dukes A | 8 | 12 | 3.6 (0.7–13.3) |
| B | 3 | 3 | 3.0 (1.2–6.6) |
| C | 4 | 8 | 17.6 (3.4–70.8) |
| D | 4 | 7 | 7.2 (3.5–15.8) |
| Right hemicolon | 6 | 11 | 17.1 (6.6–70.8) |
| Left hemicolon | 8 | 11 | 4.5 (1.7–13.3) |
| Rectum | 5 | 8 | 2.6 (0.7–5.6) |

[a]Geometric mean (and range)
A+B *vs* C+D; $P<0.01$
Right hemicolon *vs* left hemicolon+rectum; $P<0.001$.

cancer incidence after removing neoplastic polyps in repeated sigmoidoscopies. Sigmoidoscopy alone is not justified in colorectal cancer screening, however, because it can reach only about half of all colorectal malignancies. Colonoscopy is too cumbersome, time consuming and costly for screening purposes.

Faecal occult blood tests have been used extensively in screening asymptomatic patients. The colorectal cancers detected have mostly belonged to Dukes A and B stages and should consequently have a better 5-year survival rate than symptomatic cases (Gnauck, 1974; Gilbertsen *et al.*, 1980; Winawer *et al.*, 1980).

Most of the current FOBTs are based on the guaiac test, giving false negative results in 9–31% and false positive results in 2–20% (Gnauck, 1974; Bassett & Goulston, 1978; Kruis *et al.*, 1979; Gilbertsen *et al.*, 1980; Winawer *et al.*, 1980, 1982; Doran & Hardcastle, 1982; Macrae & St. John, 1982. The combined Fecatwin S-FECA-EIA kit aims at eliminating all false positive results. Because the FECA-EIA test is comparatively expensive, it is suggested that only the Fecatwin S positive samples should be investigated with the FECA-EIA test. However, this will lead to some false negative test results, which in our cancer material would have amounted to 13% of the single tests because 10% of the samples were negative with Fecatwin S and 3% negative with FECA-EIA. Fecatest gave a corresponding false negative rate of 7% and both the sensitive tests had a sensitivity limit as low as 1.2–1.7 mg Hb $g^{-1}$ faeces. There seems to be no significant difference in sensitivity between Fecatwin S and the original Fecatest (Adlercreutz *et al.*, 1982). Haemoccult and Fecatwin were found to be much less sensitive, giving 23% false negative single test results. The difference was probably not due to homogenization of the faeces, which was necessary in order to get comparable results, because in a previous study the difference was larger when non-homogenized samples were studied (Adlercreutz *et al.*, 1982 and in press). Quite recently Doran *et al.* (1982) reported a 30% false negative rate for Haemoccult using the standard 3-day regimen. False negative results with Haemoccult still occurred in 10% after a 6-day test period. The most sensitive FOBTs all need a very strict peroxidase-free diet (Gnauck, 1974; Morris *et al.*, 1976; Adlercreutz *et al.*, 1978; Macrae *et al.*, 1982). Because dietary restrictions seem to be difficult for patients to follow, even under controlled circumstances, as in our study, the human haemoglobin specific test appears to be mandatory in colorectal cancer screening.

Anaemia and melaena are common clinical signs of cancer of the right hemicolon. In the present study the faecal samples from patients with cancer in the right hemicolon were found to contain more blood than those from patients with cancer in the left hemicolon and rectum. A statistically significant correlation between the daily blood loss and the tumour stage was also observed. Similar results have been reported previously by Macrae *et al.* (1982).

In a trial by Songster *et al.* (1980) 29% of the colorectal cancer patients did not show bleeding by either the relatively insensitive immunological method of Barrows *et al.* (1976) or the Haemoccult II test when one or more faecal specimens were tested. In their survey, the immunological test proved more sensitive than the Haemoccult II test, especially in bleeding from the colon. For rectal bleeding, however, their immunological test was positive in only 50% of the patients. Using a more sensitive version of this test, Williams *et al.* (1982) showed that all cancer patients had detectable Hb in their stools but they did not quantify the daily bleeding. This is in agreement with our results, which indicated bleeding in 97% of the cancer patients as judged by FECA-EIA alone and in 100% if both Fecatwin S and FECA-EIA were performed separately. Williams *et al.* (1982) also revealed that the specific test was more sensitive than the Haemoccult II test, as shown in the present study also. Rosenfield *et al.* (1978) reported a specific test based on the inhibition of anti-Rh 29 by erythrocytic stroma in faeces. The sensitivity of this test was equivalent to that of the Haemoccult II test.

In the present study 20% of the cancer patients had a blood loss of <2.7 mg Hb $g^{-1}$ faeces. In these patients with slight bleeding, FECA-EIA was the only test giving 100% positive results. Measured with the $^{51}Cr$ method the sensitivity limit for FECA-EIA was 0.7 mg Hb $g^{-1}$ faeces. However, this is below the true sensitivity limit of the $^{51}Cr$ method because at the level most of the radioactivity is due to biliary excretion of $^{51}Cr$ (Stephens & Lawrenson, 1969). Thus, *in vivo* sensitivity of FECA-EIA cannot be determined exactly because no sufficiently sensitive reference method is available. *In vitro* the FECA-EIA test detects 0.01–0.05 mg Hb added to 1 g of faeces. The immunological test therefore seems to be at least 10 times more sensitive than the guaiac screening test.

The test used for screening, in this case Fecatwin S, must be as sensitive as possible in order not to give false negative test results. This guaiac test is sensitive, but it was still negative in 10% of the samples (10.5% of the colorectal cancer patients) when testing only one or two samples from each subject. Despite this, we still recommend the use of the combined Fecatwin S and FECA-EIA kit and not the FECA-EIA test alone for screening because FECA-EIA, being a non-isotopic immunoassay, is relatively expensive. Using Fecatwin S as a screening test, only ~5–15% of the samples need to be retested with FECA-EIA, depending on the diet of the population screened. The present study was not aimed at investigating the usefulness of the combined kit for screening purposes, but at comparing the test with a reference method in normal subjects and in colorectal cancer patients, and at determining its sensitivity and accuracy. In our hands the immunological test proved to be both accurate and sensitive. We suggest that three consecutive samples be taken for screening purposes, because in this way the percentage of false negative results shown by Fecatwin S and caused by non-uniform distribution of blood in the faeces (Rosenfield *et al.*, 1978) will probably be reduced to <5%.

It is concluded that the new two-phase test for occult blood in faeces is the first practical kit available and has great potential in colorectal cancer screening. No dietary restrictions are needed, which will increase patient compliance. Used in the suggested way, the test will probably lead to a good cost-benefit ratio. Further studies under field conditions are necessary before a final evaluation of this test is possible.

Labsystems Corp. supplied the Fecatwin sensitive FECA-EIA kits.

The study was supported by grants from the Medical Research Council of the Academy of Finland (H. Adlercreutz) and the Finnish Cancer Society (M.J. Turunen).

### References

ADAMS, E.E. & LAYMAN, K.M. (1974). Immunochemical confirmation of gastrointestinal bleeding. *Ann. Clin. Lab. Sci.*, **4**, 343.

ADLERCREUTZ, H., LIEWENDAHL, K. & VIRKOLA, P. (1978). Evaluation of Fecatest, a new guaiac test for occult blood in feces. *Clin. Chem.*, **24**, 756.

ADLERCREUTZ, H., LIEWENDAHL, K. VIRKOLA, P. & TURUNEN, M. (1982). Discrepancy in results from three guaiacum resin tests. *Br. Med. J.*, **284**, 1118.

BARROWS, G.H., BURTON, R.M., JARRETT, D.D., RUSSEL, C.G., ALFORD, A.D. & SONGSTER, C.L. (1976). Immunochemical detection of human blood in feces. *Am. J. Clin. Pathol*, **69**, 342.

BASSETT, M.L. & GOULSTON, K.J. (1978). Colorectal cancer: the challenge of early detection. *Med. J. Aust.*, **1**, 489.

BASSETT, M.L. & GOULSTON, K.J. (1980). False positive and negative Hemoccult reactions on a normal diet and effect of diet restriction. *Aust. N.Z. J. Med.*, **10**, 1.

BLUM, U., COPPEL, J. & UNGEHEUER, E. (1983). Effektivität der Krebs-Vorsorgeuntersuchung bei der Früherfassung kolorektaler Karzinome. *Dtsch Ärtzebl.*, **28**, 29.

DAVIES, J.W.L. (1971). Blood volume studies. In: *Radioisotopes Medical Diagnosis*, pp. 336. (Eds. Belcher & Vetter) London: Butterworths.

DUKES, C.E. (1932). The classification of cancer of the rectum. *J. Pathol. Bacteriol.*, **35**, 323.

DORAN, J. & HARDCASTLE, J.D. (1982). Bleeding pattern in colorectal cancer: The effect of aspirin and the implications for faecal occult blood testing. *Br. Med. J.*, **69**, 711.

ENGVALL, E. & PERLMANN, P. (1971). Enzyme-linked immunosorbent assay (ELISA). Quantitative assay of immunoglobulin. *C. Immunochemistry*, **8**, 871.

FRIEDMAN, B.I. (1972). Radionuclide determination of gastrointestinal blood loss. *Semin. Nucl. Med.*, **2**, 265.

GILBERTSEN, V.A. (1974). Proctosigmoidoscopy and polypectomy in reducing the incidence of rectal cancer. *Cancer*, **34**, 936.

GILBERTSEN, V.A., MCHUGH, R., SCHUMAN, L. & WILLIAMS, S.E. (1980). The early detection of colorectal cancers. A preliminary report of the results of the occult blood study. *Cancer*, **45**, 2899.

GNAUCK, R. (1974). Okkultes Blunt in Stuhl als Suchtest nach colorectalem Krebs und prekanzerösen Polypen. *Zschr. Gastroenterol.*, **12**, 239.

GOULSTON, K. & DAVIDSON, P. (1980). Fecal occult blood testing in patients with colonic symptoms. *Med. J. Aust.*, **2**, 667.

GREEGOR, D.H. (1967). Diagnosis of large bowel cancer in the asymptomatic patients. JAMA, **201**, 123.

HEINRICH, H.C. (1982). Sensitivität und Spezifität chemischerimmunochemischer und nuklearmedizinischer Methoden zum Nachweis und zur Quantifizierung okkulter gastrointestinaler Blutverluste. *Internist Prax.*, **22**, 665.

ILLINGWORTH, D.G. (1965). Influence of diet on occult blood tests. *Gut*, **6**, 595.

KRUIS, W., WIENZIERL, M. & EISENBURG, J. (1979). Endoskopische Diagnosen bei positivem und negativem Haemoccult-Test. *Med. Klin.*, **74**, 1641.

MACRAE, F.A. & ST. JOHN, J.B. (1982). Relationship between pattern of bleeding and Hemoccult sensitivity in patients with colorectal cancers or adenomas. *Gastroenterology*, **82**, 891.

MACRAE, F.A., ST. JOHN, J.B., CALIGIORE, P., TAYLOR L.S. & LEGGE, J.W. (1982). Optimal dietary conditions for Haemoccult testing. *Gastroenterology*, **82**, 899.

MORRIS, D.W., HANSELL, J.R., OSTROW, J.D., LEE, C.-S. & WEIGAND, P. (1976). Reliability of chemical tests for faecal occult blood in hospitalized patients. *Am. J. Dig. Dis.*, **21**, 845.

ROSENFIELD, R.E., KOCHWA, S., KACZERA, Z. & MAIMON, J. (1978). Nonuniform distribution of occult blood in feces. *Am J. Clin. Pathol.*, **71**, 201.

SONGSTER, C.L., BARROWS, G.H. & JARRETT, D.D. (1980). Immunochemical detection of fecal occult blood. The fecal smear punch-disc test: A new noninvasive screening for colorectal cancer. *Cancer*, **45**, 1099.

STEPHENS, F.O. & LAWRENSON, K.B. (1969). $^{51}Cr$ excretion in bile, *Lancet*, **i**, 158.

TURNBULL, P.R. JR., KYLE, K., WATSON, F.R. & SPRATT, J. (1967). Cancer of the colon: The influence of the no-touch isolation technic on survival rates. *Ann. Surg.*, **166**, 420.

VELLACOTT, K.D., BALDWIN, R.W. & HARDCASTLE, J.D. (1981). An immunofluorescent test for fecal occult blood. *Lancet*, **i**, 18.

WIENER, S.L. & WIENER, J. (1975). Red fruit causing false positive occult blood tests in stools. *N. Engl. J. Med.*, **292**, 408.

WILLIAMS, J.A.R., HUNTER, R., SMITH, M., COLES, M.E., HUBERT, T.W. & THOMAS, D.W. (1982). Screening for colorectal cancer. Evaluation of an immunological test for occult bleeding from colorectal neoplasia. *Aust. N.Z. J. Surg.*, **52**, 617.

WINAWER, S.J., ANDREWS, M., FLEHINGER, P. SHERLOCK, P., SCHOTTENFELD, D. & MILLER, D.G. (1980). Progress report on controlled trial of fecal occult blood testing for the detection of colorectal neoplasia. *Cancer*, **45**, 2959.

WINAWER, S.J., FLEISHER, M., BALDWIN, M. & SHERLOCK, P. (1982). Current status of fecal occult blood testing in screening for colorectal cancer, CA.-A. *Cancer J. Clinicians*, **32**, 100.

WINAWER, S.J., LEIDNER, S.D., HAJDU, S.I. & SHERLOCK, P. (1978). Colonoscopic biopsy and cytology in the diagnosis of colon cancer. *Cancer*, **42**, 2849.